# Cultural adaptation and harmonization of four Nordic translations of the revised Premature Infant Pain Profile (PIPP-R)

**DOI:** 10.1186/s12887-018-1322-5

**Published:** 2018-11-08

**Authors:** Emma Olsson, Agneta Anderzén-Carlsson, Sigríður María Atladóttir, Anna Axelin, Marsha Campbell-Yeo, Mats Eriksson, Guðrún Kristjánsdóttir, Emilia Peltonen, Bonnie Stevens, Bente Vederhus, Randi Dovland Andersen

**Affiliations:** 1 Department of Pediatrics, Faculty of Medicine and Health, Örebro University Hospital, S-701 85 Örebro, Sweden; 2 Faculty of Medicine and Health, School of Medical Sciences, Örebro University, Örebro, Sweden; 3 University Health Care Research Center, Faculty of Medicine and Health, Örebro University, Örebro, Sweden; 4 Faculty of Nursing, University of Iceland, Reykjavik, Iceland; 5 Neonatal Intensive Care Unit, Lanspitali University Children’s Hospital, Reykjavik, Iceland; 6 Department of Nursing Science, University of Turku, Turku, Finland; 7 School of Nursing, Faculty of Health Professions and Departments of Pediatrics, Psychology & Neuroscience, Dalhousie University, Halifax, Canada; 8 Centre for Pediatric Pain Research, IWK Health Centre, Halifax, Canada; 9 Faculty of Medicine and Health, School of Health Sciences, Örebro University, Örebro, Sweden; 10 Lawrence S Bloomberg, Faculty of Nursing, University of Toronto, Toronto, Canada; 11 Department of Nursing, The Hospital for Sick Children, Toronto, Canada; 12 Department of Pediatrics, Haukeland University Hospital, Bergen, Norway; 13 Department of Child and Adolescent Health Services, Telemark Hospital, Skien, Norway; 14 Department of Neurobiology, Care Sciences and Society, Karolinska Institutet, Stockholm, Sweden

**Keywords:** Neonatal, Pain, Pain assessment

## Abstract

**Background:**

Preterm infants are especially vulnerable to pain. The intensive treatment often necessary for their survival unfortunately includes many painful interventions and procedures. Untreated pain can lead to both short- and long-term negative effects. The challenge of accurately detecting pain has been cited as a major reason for lack of pain management in these non-verbal patients. The Premature Infant Pain Profile (PIPP) is one of the most extensively validated measures for assessing procedural pain in premature infants. A revised version, PIPP-R, was recently published and is reported to be more user-friendly and precise than the original version. The aims of the study were to develop translated versions of the PIPP-R in Finnish, Icelandic, Norwegian, and Swedish languages, and to establish their content validity through a cultural adaptation process using cognitive interviews.

**Methods:**

PIPP-R was translated using the recommendations from the International Society for Pharmacoeconomics and Outcomes Research and enhanced with cognitive interviews. The respondent nurse was given a copy of the translated, national version of the measure and used this together with a text describing the infant in the film to assess the pain of an infant in a short film. During the assessment the nurse was asked to verbalize her thought process (thinking aloud) and upon completion the interviewer administered probing questions (verbal probing) from a structured interview guide. The interviews were recorded, transcribed, and analyzed using a structured matrix approach.

**Results:**

The systematic approach resulted in translated and culturally adapted versions of PIPP-R in the Finnish, Icelandic, Norwegian and Swedish languages. During the cultural adaptation process several problems were discovered regarding how the respondent understood and utilized the measure. The problems were either measure problems or other problems. Measure problems were solved by a change in the translated versions of the measure, while for other problems different solutions such as education or training were suggested.

**Conclusions:**

This study have resulted in translations of the PIPP-R that have content validity, high degree of clinical utility and displayed beginning equivalence with each other and the original version of the measure.

## Background

Preterm infants, delivered weeks and often months early, are especially vulnerable to pain. All their bodily systems, including the nervous system, are immature. While their pain-signaling pathways are present and fully functional, their pain inhibitory systems are still underdeveloped, causing their pain to be prolonged and increased [1]. The intensive treatment often necessary for their survival includes many painful interventions and procedures. A recent Dutch study reported that infants in the neonatal intensive care unit (NICU) underwent a mean of 11.4 (SD 5.7) painful procedures per day [2], findings consistent with a recent systematic review of epidemiological studies [3]. Sadly, pain-relieving interventions were associated with fewer than half of these procedures [3].

The challenges of accurately detecting pain in these non-verbal patients has been cited as a major reason for lack of pain management in this population [4]. While over 40 infant pain measures have been published, their validity varies widely, adding to the difficulties of accurate pain assessment, especially in preterm infants [5]. Clinical use of insufficiently validated measures poses a risk to patient safety as they may result in both under- and over-assessment of pain. Under-assessment may cause unnecessary pain and suffering, as untreated pain in an infant can lead to both short- and long-term negative consequences including physiologic instability and altered development of the neurological, somatosensory and stress response systems [6] and poorer brain development [7]. Repeated exposure to pain may also lower the infants’ pain thresholds and increase sensitivity to subsequent pain [1] an effect that can persist after the neonatal period [8–10].

Over-assessment of pain, i.e. assuming that the patient is in pain while he/she is not, may lead to unnecessary use of pain-relieving medication with their potentially negative side effects [11]. Pharmacological treatments should be used selectively during the neonatal period because of the infants’ immature drug metabolism and elimination. The use of opioids increases the risk of respiratory depression and may also affect neurodevelopment [12, 13]. These vulnerabilities emphasize the importance of valid and effective assessment of pain in this patient group in order to both minimize pain and the risks associated with pharmacological treatment of pain [14, 15].

The Premature Infant Pain Profile (PIPP) is one of the most extensively validated measures for assessing procedural pain in premature infants [15, 16]. PIPP is currently being used in clinical practice in several Nordic NICUs. A revised version, PIPP-R, was recently published and is reported to be more user-friendly and precise than the original version [17, 18].

In accordance with the COSMIN taxonomy [19] face and content validity are two aspects of content validity. Both face and content validity are judgment-based, qualitative evaluations. While face validity concerns whether the PIPP/PIPP-R looks like a good reflection of the construct pain, construct validity is an evaluation of whether the PIPP/PIPP-R is an adequate representation of the construct pain concerning relevance and comprehensiveness. Content validity of the PIPP/PIPP-R was established during the construction of the PIPP measure [16]. In addition, content validity also needs to be addressed for all translated versions of the measure, as their validity is dependent upon how the translation and cultural adaptation were carried out [20]. Content validity should be assessed by those who are going to use the scale [21], for example through cognitive interviews where future users of the scale explain their understanding and use of the measure [20]. When establishing content validity of a translated measure, the translation needs to maintain fidelity towards the original version [22]. A systematic cultural adaptation ensures that the original meaning and content is retained in the translated versions of the measure [23]. Measure equivalence is a prerequisite for valid comparisons between data collected with different language versions of a measure [24]. Performing a parallel and collaborative translation and cultural adaptation of several language version of the measure simultaneously helps ensure beginning equivalence across the translated versions and between the translated versions and the original version. In that regard, this collaborative process will support future collaborative research involving the PIPP-R.

Translation and cultural adaptation is a necessary first step towards clinical implementation of the revised version of the PIPP in the Nordic countries. As such, the aims of this study were to develop translated versions of the PIPP-R in Finnish, Icelandic, Norwegian, and Swedish languages, and to establish their content validity and beginning equivalence through a cultural adaptation process using cognitive interviews.

## Methods

### Study design

**Fig. 1:**
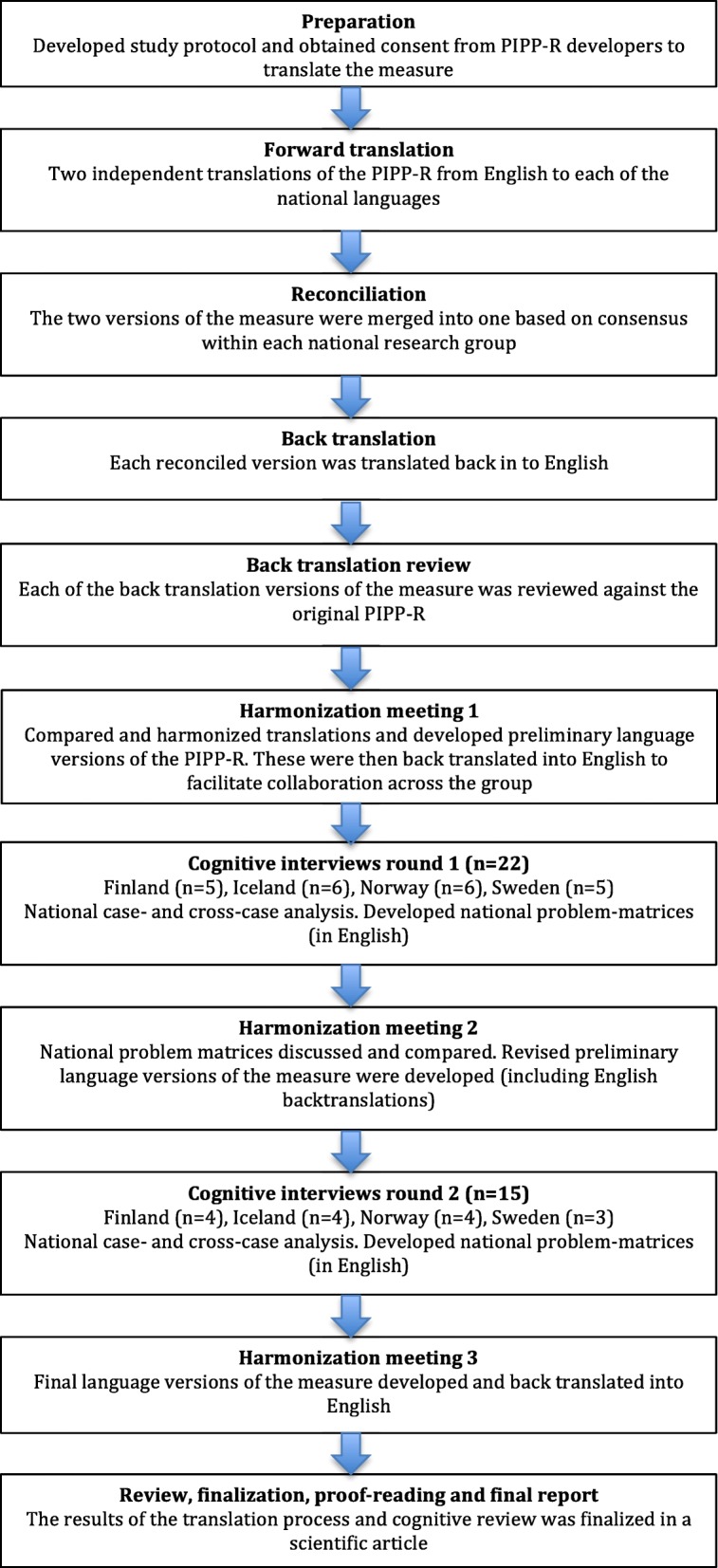
The translation and cultural adaptation process based on Wild et al. 2005 as described in Andersen et al. 2015

The study followed the methodology recommended by the International Society For Pharmacoeconomics and Outcomes Research (ISPOR) Task Force for Translation and Cultural Adaptation [20]. An existing study protocol developed based on ISPOR methodology and enhanced with cognitive interviews [25] was modified for this study. For an overview of the translation and cultural adaptation process, see Fig 1.

The authors of the original PIPP-R granted permission to translate PIPP-R and the work was done in collaboration with them.

### PIPP-R

PIPP-R is a multidimensional pain assessment measure. It consists of three behavioral indicators (brow bulge, eye squeeze, and naso-labial furrow), two physiological indicators (heart rate and oxygen saturation), and two contextual indicators (corrected gestational age and behavioral state) that modify the score. The assessment starts with a 15 s baseline measurement of the heart rate and oxygen saturation and the infants’ corrected gestational age and behavioral state are noted. Changes in physiological and behavioral indicators are then assessed during the first 30 s of the painful procedure. If there is a response in the physiologic and behavioral variables during the procedure, scores for corrected gestational age and behavioral state are added. These contextual variables weight greater points for the more immature infants and the infants in quiet sleep state since it is well known that these infants react with less vigorous pain cues [18]. Each indicator is scored from zero to three points and summed into a total pain intensity score ranging from zero to 21 points.

### Translation procedure

The process, which was performed during 2015, started with a forward translation in which two independent translations of the PIPP-R were performed in each country, one by the national investigator with extensive knowledge of neonatal pain assessment and one by a certified translator. The two versions of the measure were then merged into one, through consensus within each national group. The respective reconciled versions were then back-translated by a native English-speaking health care professional or a certified translator blinded to the original scale. All translators were bilingual and translated into their native language. Each back-translated version of the measure was reviewed against the original PIPP-R, any discrepancies examined against the reconciled version, and appropriate revisions were made on national level. During a meeting with all members of the group (harmonization meeting 1), the four preliminary versions of the PIPP-R were compared and harmonized with each other and with the original PIPP-R measure. Back-translated English versions of the preliminary measures were used during the meetings to make comprehension and comparison possible within the research group.

### Cognitive interviews

The cultural adaptation of the national versions of the PIPP-R was carried out using cognitive interviews as described by Andersen et al. [25]. Originally, cognitive interviewing aims to understand how respondents understand, process and answer questions and also to identify potential problems with survey questions. The process often starts with the respondents “thinking aloud,” recounting everything they think about while completing a task. The investigator can also use verbal probing, asking the respondents to paraphrase questions, define meanings of different items, or explain their answers [26]. Andersen and colleagues (2014) modified this approach for use with observational pain scales.

A structured interview guide was developed for the cognitive interviews by two of the national researchers (EO and RDA). It included training of the participant in the think-aloud method and an introduction to the PIPP-R, followed by a section where the respondent used the think-aloud method to describe their understanding of the PIPP-R measure and a set of structured questions about how the respondent understood each item of the PIPP-R. Finally, the respondent was given the opportunity to add anything that had not been brought up earlier. The same national researchers (EO and RDA) each carried out one pilot interview to test the interview guide and small adjustments were made. The interview guide was then translated into the different languages by the national investigators in each country.

### Sample and setting

A total of 37 nurses with a minimum of one year of NICU experience and fluent in the target national language were recruited and agreed to be interviewed. A purposeful sampling procedure was used to include a diverse group of nurses in regard to age, education, clinical experience, and experience with structured pain assessment measures. The participating nurses had between 1 and 33 years of NICU experience, and 15 of them had a specialist education. While all had access to pain assessment measures at their unit, just over half reported using them (see Table 1). None of them had previous experience using PIPP-R.

**Table 1:** Demographic information about the included nurses

Participants in each country (round 1+ round 2)	Finland *n* = 9 (5 + 4)	Iceland *n* = 10 (6 + 4)	Norway (n = 10) (6 + 4)	Sweden (*n* = 8) (5 + 3)
Level of education	RN – 7 Specialist nurse - 2	RN – 6 Specialist nurse – 4	RN – 6 Specialist nurse - 3	RN – 2 Specialist nurse - 6
Clinical NICU experience (years)	1.5–13.5 (median 3.5)	1.5–24 (median 8.5)	1–33 (median 16.3)	2–22 (median 11.5)
Experience with pain assessment	9	9	10	8
Access to pain assessment instrument at unit	9	9	10	8
Received training in how to use instrument at unit	7	4	9	6
Use pain assessment instrument in daily work	4	0	10	8

### Data collection procedure

Following informed consent, the respondent participated in a cognitive interview that was recorded and transcribed verbatim. The respondent nurse was given a copy of the translated PIPP-R and was instructed to read it through and familiarize herself with the measure. Subsequently, the respondent was asked to use the PIPP-R to assess pain from a short film showing an infant exposed to a painful procedure (the same film was used for all respondents) and an accompanying written text describing the infant in the film. The respondent was asked to verbalize her thought process while completing the assessment (thinking aloud), and upon completion of the assessment the interviewer used probing questions from the structured interview guide (verbal probing). The aim of the interviews was to identify any problems in the understanding and application of the measure. One researcher performed the interviews in Finland, Iceland, and Sweden respectively, while two researchers performed the interviews in Norway.

Data were collected and analyzed in two rounds during 2016. The first round comprised 22 interviews (five in Finland, six in Iceland, six in Norway, and five in Sweden) and the second round 15 interviews (four in Finland, four in Iceland, four in Norway, and three in Sweden). The interviews lasted from 38 min to 66 min.

### Data analysis

The interview data were analyzed using Miles and Huberman’s [27] approach to data analysis using matrices and case- and cross-case analyses. A predefined problem matrix was developed with a set of organizational categories including the items on the PIPP-R (change in heart rate, decrease in oxygen saturation, brow bulge, eye squeeze, naso-labial furrow, corrected gestational age and behavioral state), the title of the measure, the scoring instructions, use of film and the overall use of the measure. Problems perceived by either the respondents or the interviewers during the interviews were entered in the matrix together with suggested strategies from the researchers for solving the problems.

After each round of interviews, the national data was independently analyzed in the national language by the national researcher. Each participant was considered a case and a single-case analysis was conducted after each interview. Data from national single-case problem matrices were compiled into one cross-case matrix, condensed and refined. The cross-case matrices were translated into English and the translated versions discussed in two harmonization meetings (one after each interview round) with all researchers present. After the first round of interviews, the researchers identified problems that required adjustment in the preliminary translated versions of the PIPP-R. These were discussed during the second harmonization meeting, and the developers of the measure were consulted about the proposed changes described below. Revisions were made and the revised versions of the respective measure where then used in the second round of interviews. A final cross-country, cross-case matrix was developed and provided an overview of all problems identified in the study.

## Results

This systematic approach resulted in translated and culturally adapted versions of the PIPP-R pain assessment measure in the Finnish, Icelandic, Norwegian, and Swedish languages. During the cultural adaptation process several problems were discovered regarding how the respondents understood and utilized the measure. The problems can be divided into two categories: measure problems and other problems. Measure problems were solved by a change in the translated versions of the measure, while for the other problems different solutions such as education or training were suggested. Measure problems were further divided into the two sub-categories: problems related to the original version of the measure and problems related to the translated versions of the measure. The respective problems will be described below and are visualized in Table 2.

**Table 2:** Problems and solutions found during the cultural adaptation process

Problems found	Solution	Finland	Iceland	Norway	Sweden
*Problems related to the original version of the scale*					
The application of the scale were not understood	Title clarification	X	X	X	
The word oxygen used to describe both oxygen supply and oxygen saturation	Wording changes to distinguish between SaO2 and FiO2	X	X	X	X
Scale uses both “corrected gestational age” and “gestational age” which respondents found confusing.	Changed to “corrected gestational age” throughout the scale	X	X	X	X
Unclear in step 2 which time frame that should be used for assessment	Clarification which 30 s that are to be used	X	X	X	X
The term “vital sign” is not explained in the scale	Changed to “physiological indicators”	X		X	X
In step 3 unclear when to include score for corrected GA and behavioral state	Clarifications on when these factors should be included	X	X	X	X
Unclear time frame for when to give points for additional oxygen	Clarification that it is the first 30 s that are to be assessed		X	X	X
*Problems related to the translated versions of the scale*					
Baseline not a familiar term	Change of wording			X	X
Not understanding the facial indicator “Brow Bulge”	Change of wording				X
Not understanding the facial indicator “Naso-labial furrow”	Change of wording	X	X	X	X
Explanatory word for the different categories (eg minimal, maximal) not making sense	Change of wording	X	X		
*Problems that can be solved with education and training*					
Unclear if you should assess baseline behavioral state once or several times	Education and training	X			
Unclear if it is duration or intensity of pain that is to be assessed	Education and training	X	X	X	X
Difficult to assess several parameters at the same time	Education and training	X	X	X	X
Would like a guiding instruction for the acquired pain score/a pain algorithm	Education and training	X	X	X	X
Uses mean heart rate/oxygen saturation instead of highest/lowest	Education and training		X		
The “+” sign before each scoring category is confusing	Education and training		X		
Difficulties distinguishing between the different behavioral states categories	Education and training		X	X	X
Unsure about how to assess a decrease in heart rate	Education and training			X	X
Difficult to understand how to score if fiO2 is increased	Education and training			X	
Scores for the time when the reaction occurs and not the duration of the reaction	Education and training			X	
Difficult to separate “Brow bulge” from “Eye squeeze” since they are highly correlated	Education and training			X	

The described problems were found in the language versions marked with “X”

### Problems related to the original version of the measure

Before the first interview round a few inaccuracies were discovered in the published original version of the PIPP-R [18] and these were adjusted for in all the translated versions. In the categorization of gestational age, the symbol > had been used instead of ≥, and as a result neonates born at 36 weeks’ gestation did not fit into any of the categories. In step 2 of the scoring instructions the phrase “maximal heart rate” was used, although the indicator says “change in heart rate” to allow scoring of both an increase and a decrease in heart rate. Finally, “gestational age” was missing from the equation in step 4 and was therefore added.

In the indicator “oxygen saturation,” respondents found the use of the word oxygen to describe both oxygen saturation and oxygen supply confusing. To clarify this, the abbreviation “SaO2” (oxygen saturation) was added to the indicator, and in the + 3 score box the abbreviation FiO2 (fraction of inspired oxygen) was used to indicate any additional, delivered oxygen. The original measure used both the terms “gestational age” and “corrected gestational age” to describe the infant’s age at the time of the assessment; several respondents were confused about whether this meant that the infant’s age at birth (gestational age) was to be reported or the infant’s age at the time for the procedure (corrected gestational age). “*.. I thought that gestational age meant the gestational age the baby was when it was born and not the gestational age the baby is now”* (I3). All translated versions were revised to use “corrected gestational age” consistently.

In step 2 of the scoring instructions respondents expressed uncertainty about the time frame for the assessment. “*It says here ‘after the procedure’. To my way of thinking it would be natural to score the child also during the procedure”* (N4). The phrase “observe infants for 30 seconds after the procedure” was changed into “observe infants during the first 30 seconds of the procedure”.

The term “vital signs” in the original PIPP-R was used to describe heart rate and oxygen saturation, but because behavioral state was mentioned in the same sentence, some of the respondents thought this was also included in the vital signs. “*So I guess I would count those three (heart frequency, oxygen saturation and baseline behavioral state) as vital signs”* (S3). The term “physiological indicators” was thus chosen instead of “vital signs” in all versions except the Icelandic to make it clearer that oxygen saturation and heart rate were intended. In Icelandic the term “vital signs” is used in health care and well understood and was therefor kept in it’s original form. In step 3 of the scoring instructions “score for corrected gestational age and behavioral state if the sub-total score >0,” respondents did not understand when to give points for corrected gestational age and baseline behavioral state and when not to. All versions were clarified with the instruction “calculate *only* if the sub-total score is >0.”

### Problems related to the translated versions of the measure

This sub-category comprised misunderstandings of the translation of certain words or phrases. A direct translation of the word “baseline” was not a well-known expression in Norway and Sweden so it was replaced with various, more idiomatic versions of “before the procedure.” “*I’m thinking it is often a bit difficult to know what the baseline is in comparison to when you observe and how you are supposed to – it has to be an average over some time” (N6*). The translation of the different facial indicators also proved difficult to understand in several of the countries. *“Naso-labial furrow.. What is that? Is it here?”* [Points to midline of upper lip] (I2). “Naso-labial furrow” was thus changed in all languages to a more descriptive phrase such as “furrow from nostril to corner of mouth” (Icelandic version) to enhance understanding, and “brow bulge” was changed to “frowning eye brows” in the Swedish version.

The explanatory words for assigning an indicator score for the different facial indicators were questioned in Iceland and Finland during the first round of interviews and a different set of descriptive scoring words were used in the second round. “*The option of ‘much’ seems to be missing from the indicator score. There is ‘moderate’ and then comes ‘maximal’* (F5). Abbreviations used in the original version of the measure, (e.g. BS for behavioral state) did not have equivalents in the target languages and were written out instead “*My first question would be what are the GA and BS indicator scores?”* (F1).

In the second round of interviews there were notably fewer measure problems and the only issue that led to a change was one nurse’s belief that she should give points for any additional oxygen given at any time before or during a procedure even if the procedure lasted longer than the 30-s assessment period. The scoring instructions for step 2 “if an infant requires an increase in oxygen at any time before or during the procedure” did not specify the time frame and was clarified to read “before or during *the first 30 seconds* of the procedure”.

### Problems that can be solved with education and training

All other issues identified did not require a change in the measure and will most likely be solved with appropriate education, training and access to a scoring manual. Some of these problems differed between countries, while some were apparent in all versions. For example, many of the respondents were conflicted about whether it was the duration or the intensity of pain that should be assessed and reported. PIPP-R uses both a descriptive word for pain in each scoring category for the facial indicators (none, minimal, moderate, or maximal) but also a time reference (< 3, 3–10, 6–8, or > 8), which was confusing for most of the respondents. “*I am having a bit of trouble with the seconds. How long it [the reaction] is actually present. But maybe rather how strong the reaction is”* (S6). Respondents from all countries acknowledged having difficulty with assessing multiple indicators at the same time, something that will probably become easier with education and training. Nurses also wanted to know what to do with the score and asked for a pain management algorithm (i.e. what score represents pain and when should pain-relieving treatments be used).

## Discussion

We translated and culturally adapted the PIPP-R pain assessment measure to Finnish, Icelandic, Norwegian, and Swedish through an international collaboration. During the translation we discovered that the respondents had a range of different problems understanding the measure as intended. Some problems were related to the original version of the measure and most were solved by clarifying the different aspects of the measure that were not understood correctly. The problems related to the translations of the measure mainly were unfamiliar words or phrases not commonly used in the various national settings. The use of different words and phrases elucidated these issues. This highlights the importance of not only translating a measure but also using a thorough translation and cultural adaptation process to preserve the meaning of the items in the measure [23, 28]. A simple direct translation procedure would not have sufficiently addressed the linguistic and cultural differences that were discovered during the work in this study.

This project was done in collaboration with the developers of the original measure [18]. This made it possible to go back to them for information about the intended use of the PIPP-R when questions arose. The PIPP-R, as well as any pain assessment measure, should be accompanied by adequate education and training before it is incorporated into clinical practice; research has shown that education can improve the use of pain assessment measures [29]. Many of the issues identified in this study will be eliminated through training and consistent education before the translated versions of the measure are used in clinical settings. A great deal of discussion was generated by the information in the scoring boxes for the facial indicators. The seemingly conflicting instructions of both intensity and duration of pain lead to discussions in the group about possibly removing the describing word from the measure. In consultation with the intention of the original measure, to be used both in clinical and research settings, this was considered as too much of a change to the published PIPP-R. A systematic on-line learning program has been developed for the PIPP-R that will probably solve this and many other issues.

A strength of this study was the simultaneous translation and harmonization process where the four translations of the PIPP-R were harmonized with both the original version of the measure [18] and with each other. Some of the problems we found were apparent in several of the translated versions, making it more likely that those problems were related to the original version of the measure and not a result of the translation. Through systematic comparisons across the different translated versions and between the translated versions and the original version we have laid the groundwork for further equivalence testing between scores obtained with the different language versions of the PIPP-R, an important requirement when these measures are used in research across countries [24].

The collaborative process resulted in beneficial discussions and diverse views of the different problems. The results of collaborative research may be more robust because of the different strengths and specialties in the group [30]. Having a team working together in different countries and with different native languages could be demanding but potential problems were reduced having English as a common language and by frequent meetings online and in person throughout the process. The results of this study are based on a rigorous translation and cultural adaptation process, which included cognitive nterviews with a total of 37 neonatal nurses with a wide variety of experience. All of them had access to pain assessment measures, but none had previous experience with the PIPP-R. This made it possible for them to have an unbiased opinion about the measure and few preconceived assumptions. Although all the nurses had access to a pain assessment measure, just over half of them reported using it in their daily practice. This is worrying because the use of pain assessment measures in this sensitive and non-verbal population is highly recommended [6]. This reported percentage might also be an over-estimation because of potential response bias [31]; nurses might have wished to create a positive impression by stating that they used pain assessment measures more frequently than they actually did. The PIPP-R was designed to enhance feasibility and feasible measures are more likely to be used [18]. We believe this study have resulted in translations of the PIPP-R with good content validity, good feasibility and beginning equivalence to the original version of the measure, all of which support the clinical utility of the measure. Assumptions regarding equivalence and clinical utility of the measure need to be tested in further studies.

While considerable time has been spent on the development of pain assessment measures to assess infant pain, less emphasis has been placed on the clinical utility of these tools. This oversight may have contributed to the lack of consistent pain assessment and management and the wide variation in practice uptake across neonatal units worldwide. Future work in this area should ensure that emphasis is placed not only on ensuring the validity and reliability of pain assessment tools but also their clinical utility. Ensuring measures are systematically translated; including appropriate evaluation is an important component that should not be overlooked. Efforts should be made to translate and culturally adapt the learning program for PIPP-R so that health care professionals will be able to use the translated versions of the measure with appropriate comprehension and knowledge of how to use the measure. Future studies should also conduct psychometric testing and cross-cultural equivalence testing of the translated versions of the measure.

Why nurses choose not to use available pain assessment measures in their daily practice is another important area that requires deeper understanding.

## Conclusions

This study have resulted in translations of the PIPP-R that have content validity, high degree of clinical utility and displayed beginning equivalence with each other and to the original version of the measure.

## Abbreviations

FiO2: Fraction of Inspired Oxygen
ISPOR: International Society for Pharmacoeconomics and Outcomes research
NICU: Neonatal Intensive Care Unit
PIPP: Premature Infant Pain Profile
PIPP-R: Premature Infant Pain profile - revised
SaO2: Oxygen saturation
